# Supplementary material for: Host Polyunsaturated Fatty Acids Potentiate Aminoglycoside Killing of Staphylococcus aureus
Source: Microbiol Spectr. 2022 Apr 4;10(2):e02767-21. doi: 10.1128/spectrum.02767-21 (PMC9045252; doi:10.1128/spectrum.02767-21)
Supplement: SUPPLEMENTAL FILE 1 — Supplemental material. Download SPECTRUM02767-21_Supp_1_seq3.pdf, PDF file, 0.4 MB [file spectrum02767-21_supp_1_seq3.pdf]

Host polyunsaturated fatty acids potentiate aminoglycoside killing of *Staphylococcus aureus*

William N. Beavers[a,b], Matthew J. Munneke[a], Alex R. Stackhouse[b], Jeffrey A. Freiberg[a,c], Eric P. Skaar[a,d,e,#]

[a]Department of Pathology, Microbiology, and Immunology, Vanderbilt University Medical Center, Nashville, Tennessee, USA

[b]Department of Pathobiological Sciences, Louisiana State University and Agricultural and Mechanical College, School of Veterinary Medicine, Baton Rouge, Louisiana, USA

[c]Division of Infectious Diseases, Department of Medicine, Vanderbilt University Medical Center, Nashville, Tennessee, USA

[d]Vanderbilt Institute for Infection, Immunology, and Inflammation, Vanderbilt University Medical Center, Nashville, Tennessee, USA

[e]Vanderbilt Institute for Chemical Biology, Vanderbilt University, Nashville, Tennessee, USA

Running Title: PUFA synergize with aminoglycosides to kill *S. aureus*

[#]Address correspondence to:

Eric P. Skaar, Ph.D., M.P.H.

Vanderbilt University Medical Center

Department of Pathology, Microbiology, and Immunology

MCN A-5301

Nashville, TN 37232

Phone: 615-343-0002

Fax: 615-343-7492

E-mail: eric.skaar@vumc.org

Figures

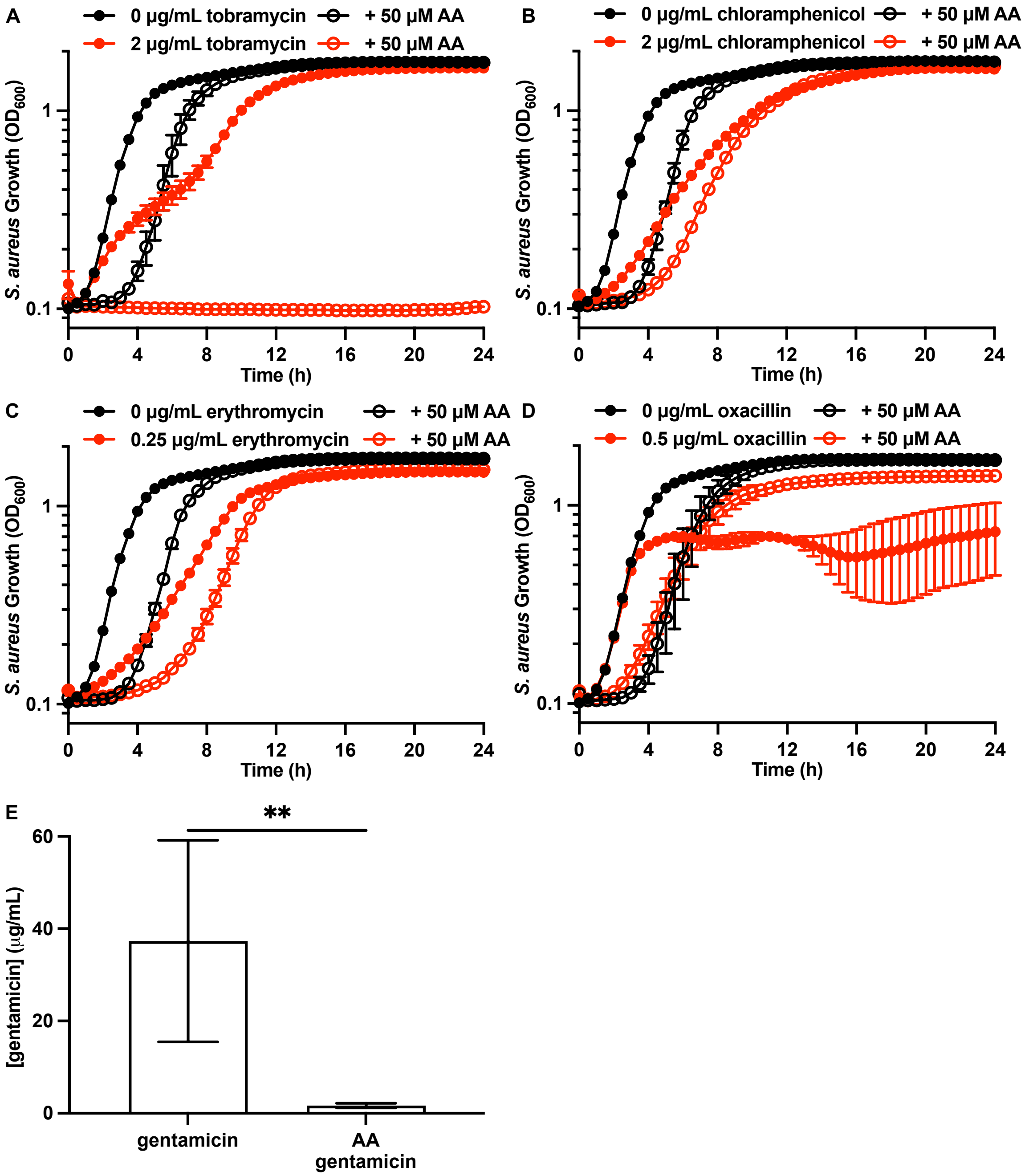

Figure S1. AA synergizes with aminoglycosides, but not other antibiotics to kill *S. aureus*. JE2 was treated with vehicle, 50 µM AA, **A.** 2 µg/mL tobramycin **B.** 2 µg/mL chloramphenicol **C.** 0.25 µg/mL erythromycin **D.** 0.5 µg/mL oxacillin, or antibiotic + AA. Bacterial growth was monitored by optical density

at 600 nm every 30 min for 24 h. Data are mean +/- standard error of the mean for measurements acquired in biological triplicate. **E.** JE2 was treated with varying concentrations of gentamicin +/- 50 μM AA for 3 h at 37 °C. After incubation, viable bacteria were quantified by dilution plating on solid medium. The lowest concentration of gentamicin with no viable bacteria is graphed for each replicate to determine the minimum inhibitory concentration. Data are presented as means +/- standard deviation of six biological replicates. *P* values were calculated by t-test where ** = $P < 0.01$.

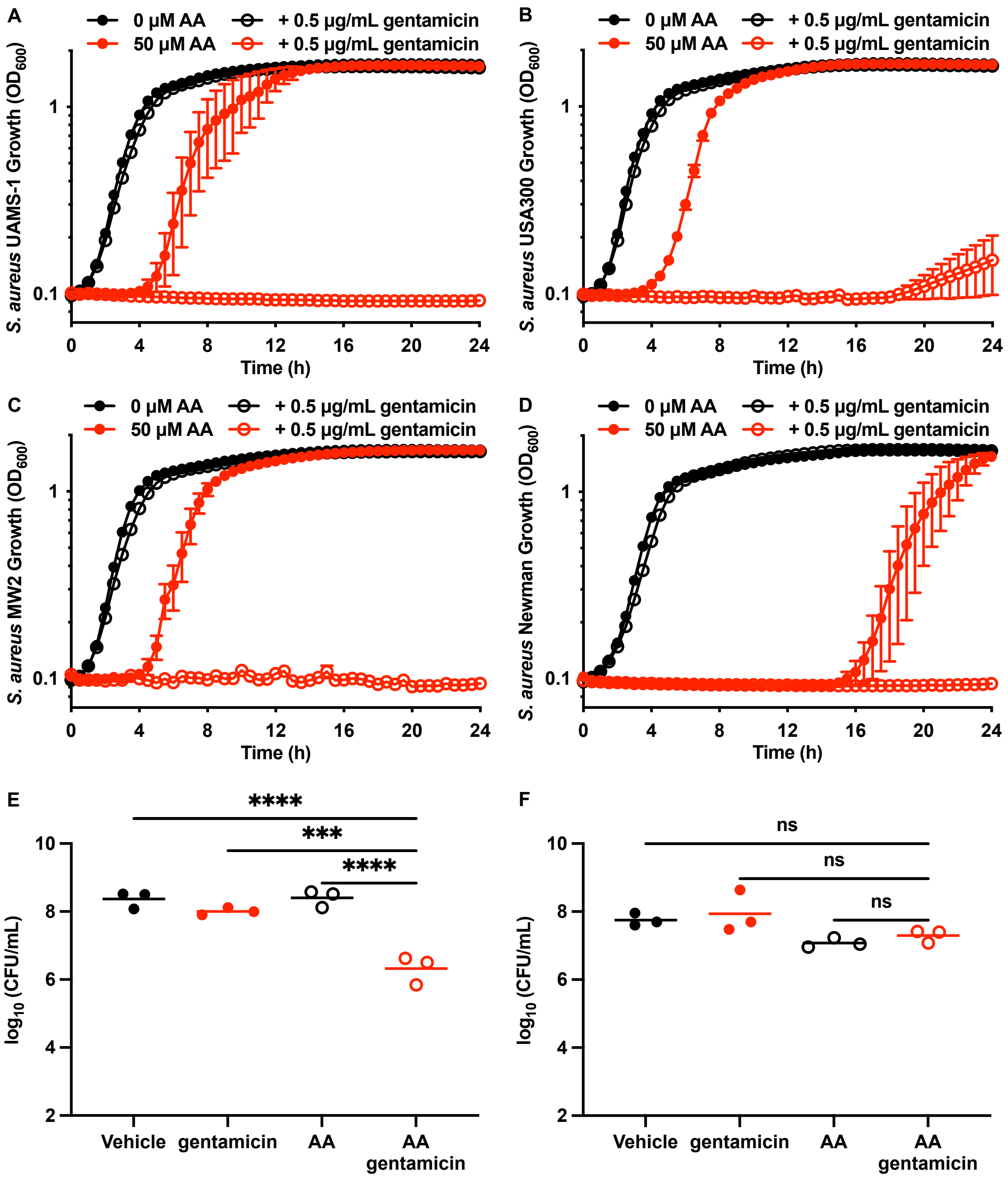

Figure S2. AA synergizes with gentamicin to kill most *S. aureus* strains tested. A. UAMS-1, **B.** USA300, **C.** MW2, or **D.** Newman was treated with vehicle, 50 μM AA, 0.5 μg/mL gentamicin, or gentamicin + AA. Bacterial growth was monitored by optical density at 600 nm every 30 min for 24 h.

Data are mean +/- standard error of the mean for measurements acquired in biological triplicate. **E.** MRSA 5005, an *S. aureus* clinical isolate susceptible to gentamicin, was treated with vehicle, 1 μg/mL gentamicin, 50 μM AA, or gentamicin + AA for 3 h at 37 °C. After incubation, viable bacteria were quantified by dilution plating on solid medium. **F.** MRSA 10554, an *S. aureus* clinical isolate resistant to gentamicin, was treated with vehicle, 1 μg/mL gentamicin, 50 μM AA, or gentamicin + AA for 3 h at 37 °C. After incubation, viable bacteria were quantified by dilution plating on solid medium. Data are presented as means, and each point represents a single biological replicate. *P* values were calculated by One-Way ANOVA where ns = $P > 0.05$, *** = $P < 0.001$, and **** = $P < 0.0001$.

Materials and Methods

Materials. *S. aureus* JE2 and NE1345 were provided by the Network on Antimicrobial Resistance in *Staphylococcus aureus* (NARSA) for distribution by BEI Resources, NIAID, NIH: Nebraska Transposon Mutant Library (NTML) Genetic Toolbox, NR-48850. *S. aureus* USA300 and UAMS-1 were generous gifts from Dr. Paul Dunman. *S. aureus* Newman was a generous gift from Dr. Olaf Schneewind. *S. aureus* MW2 was a generous gift from Dr. Frank DeLeo. MRSA 5005 and MRSA 10554 are clinical *S. aureus* isolates collected from the VUMC Microbiology Laboratory with approval from the VUMC Institutional Review Board. MRSA 5005 was isolated from a blood culture collected from a patient with endocarditis. MRSA 10554 was isolated from bone tissue from a patient with osteomyelitis. Antibiotic susceptibility patterns for these isolates were initially determined as part of the routine clinical microbiology laboratory testing. The isolates were subsequently submitted for whole genome sequencing to identify the genetic basis of their respective resistance patterns. All plasticware was from USA Scientific (Ocala, FL) or Corning (Corning, NY). All *S. aureus* strains were grown on tryptic soy agar (TSA) or in tryptic soy broth (TSB) (Becton-Dickinson, Franklin Lakes, NJ). All other chemicals were obtained from Sigma (St. Louis, MO), unless stated otherwise. All data were plotted, and statistical analyses performed in Prism 9 (GraphPad, La Jolla, CA).

***S. aureus* overnight bacterial cultures.** Unless noted otherwise, *S. aureus* strains were streaked on TSA and incubated 18 h at 37 °C. Single colonies were inoculated into 5 mL of TSA in 15 mL polypropylene tubes with aeration lids. Bacterial cultures were incubated at 37 °C with orbital shaking at 180 rpm and a 45° angle for 16 h in an Innova44 shaking incubator (Eppendorf, Hauppauge, NY).

***S. aureus* kinetic growth curves.** *S. aureus* overnights were grown as described above in biological triplicate. Overnights were diluted 100X into 5 mL of TSB in 15 mL polypropylene tubes with aeration lids. Bacterial cultures were incubated at 37 °C with orbital shaking at a 45° angle and 180 rpm for 3 h. Into each well of a 96-well plate, the bacterial outgrowths were diluted 100X into 100 μL of TSB containing the stressors to be tested. For the kinetic growth curve experiments in Figure 1, the bacteria were incubated with vehicle, 1 μg/mL gentamicin, 50 μM fatty acid (arachidonic acid, palmitic acid, or linoleic acid), or fatty acid and gentamicin, as described in the respective figure legends. For the kinetic

growth curve experiments in Figure S1, the bacteria were incubated with vehicle, 50 μM arachidonic acid, 2 μg/mL tobramycin, 2 μg/mL chloramphenicol, 0.25 μg/mL erythromycin, 0.5 μg/mL oxacillin, or arachidonic acid and the respective antibiotic, as described in the respective figure legends. For the kinetic growth curve experiments in Figure 2, the bacteria were incubated with vehicle, 5 μM menadione, 15 μM arachidonic acid, 16 μg/mL gentamicin, or arachidonic acid and gentamicin, as described in the respective figure legends. For the kinetic growth curve experiments in Figure S2, the bacteria were incubated with vehicle, 0.5 μg/mL gentamicin, 50 μM arachidonic acid, or arachidonic acid and gentamicin, as described in the respective figure legends. All growth curves were performed at 37 °C with linear shaking at 567 cpm (3 mm) for 24 h, taking readings of the optical density at 600 nm every 30 min on an EPOCH 2 plate reader (Bio Tek, Winooski, VT). Data were plotted and statistical analyses were performed in Prism 9 (GraphPad, La Jolla, CA).

***S. aureus* CFU plating.** *S. aureus* overnights were grown as described above in biological triplicate. Overnights were diluted 100X into 5 mL of TSB in 15 mL polypropylene tubes with aeration lids. Bacterial cultures were incubated at 37 °C with orbital shaking at a 45° angle and 180 rpm for 3 h. Into each well of a 96-well plate, the bacterial outgrowths were diluted 100X into 100 μL of TSB containing the stressors to be tested. For the CFU experiments in Figures 1 and S2, the bacteria were incubated with vehicle, 1 μg/mL gentamicin, 50 μM fatty acid (arachidonic acid, palmitic acid, or linoleic acid), or fatty acid and gentamicin, as described in the respective figure legends. The 96-well plate was incubated at 37 °C with orbital shaking at 180 rpm for 3 h. For the CFU experiments in Figure 2, the bacteria were incubated with vehicle, 15 μM arachidonic acid, 16 μg/mL gentamicin, or arachidonic acid and gentamicin, as described in the respective figure legend. The 96-well plate was incubated at 37 °C with orbital shaking at 180 rpm for 18 h. After incubation, each well of the 96-well plate was diluted 10^0 through 10^{-6} in PBS in 10-fold intervals. Each dilution was plated (10 μL) on TSA and incubated overnight at 37 °C followed by CFU enumeration.

Gentamicin minimum inhibitory concentration of *S. aureus* determination. JE2 was treated with a range of concentrations of gentamicin (64, 32, 16, 8, 4, 2, 1, 0.5, 0.25, 0.125, 0.0625, and 0 μg/mL) or gentamicin (8, 4, 2, 1, 0.5, 0.125, 0.0625, 0.0313, 0.0156, 0.00781, 0.00391, and 0 μg/mL) + 50 μM AA

and dilution plated as described above. The lowest concentration of gentamicin where no viable bacteria were detected was graphed for each replicate to determine the minimum inhibitory concentration of gentamicin with and without AA co-treatment.
